# Three in a Box: Understanding Cardiomyocyte, Fibroblast, and Innate Immune Cell Interactions to Orchestrate Cardiac Repair Processes

**DOI:** 10.3389/fcvm.2019.00032

**Published:** 2019-04-02

**Authors:** Stelios Psarras, Dimitris Beis, Sofia Nikouli, Mary Tsikitis, Yassemi Capetanaki

**Affiliations:** ^1^Center of Basic Research, Biomedical Research Foundation of the Academy of Athens, Athens, Greece; ^2^Center of Clinical, Experimental Surgery & Translational Research, Biomedical Research Foundation of the Academy of Athens, Athens, Greece

**Keywords:** cardiac regeneration, heart failure, innate immune cells, cardiac repair, cardiomyocytes, cardiac macrophages, cardiac fibroblasts, fibrosis

## Abstract

Following an insult by both intrinsic and extrinsic pathways, complex cellular, and molecular interactions determine a successful recovery or inadequate repair of damaged tissue. The efficiency of this process is particularly important in the heart, an organ characterized by very limited regenerative and repair capacity in higher adult vertebrates. Cardiac insult is characteristically associated with fibrosis and heart failure, as a result of cardiomyocyte death, myocardial degeneration, and adverse remodeling. Recent evidence implies that resident non-cardiomyocytes, fibroblasts but also macrophages -pillars of the innate immunity- form part of the inflammatory response and decisively affect the repair process following a cardiac insult. Multiple studies in model organisms (mouse, zebrafish) of various developmental stages (adult and neonatal) combined with genetically engineered cell plasticity and differentiation intervention protocols -mainly targeting cardiac fibroblasts or progenitor cells-reveal particular roles of resident and recruited innate immune cells and their secretome in the coordination of cardiac repair. The interplay of innate immune cells with cardiac fibroblasts and cardiomyocytes is emerging as a crucial platform to help our understanding and, importantly, to allow the development of effective interventions sufficient to minimize cardiac damage and dysfunction after injury.

## Tissue Repair and Regeneration: The Crucial Cardiac Inabilities, Consequences, and Exceptions

The harmonious homeostatic balance of cellular and molecular interactions in a given tissue may be dramatically disturbed upon insult. The tissue and organ response, coordinated by local cellular and molecular players and gradually by systemic reactions, will either lead to sealing of a wound, isolation, or extinction of an infectious or toxic agent, manipulation of a stress condition etc. However, the achievement of an efficient homeostatic repair requires the ability of the parenchyma to regenerate. In humans and mammals, liver, blood, skeletal muscle and intestinal epithelium have a good regenerative ability, whereas nervous system and heart fail to do so (1, 2). This is disastrous for the cardiac tissue, particularly following a major yet common injury event such as myocardial infarction (MI). Danger signals (DAMPs) released by dying cardiomyocytes, elicit inflammatory reactions and tissue remodeling to stabilize the damaged myocardium (3). Unfortunately, the myocardium has very weak regenerative capacity because the survived cardiomyocytes practically cannot proliferate and the heart contains very few cardiomyocyte progenitors (4, 5). As a result, the primary scar may get extended and adjacent and remote regions are affected with fibrosis, a major hallmark of adverse tissue remodeling events, which influence the entire organ, leading eventually to heart failure (HF), a currently incurable public health problem. Ischemic conditions are responsible for the major part of HF cases. However, similar pathways and sequence of events may characterize the inability of the heart to cope with the rest 16 known HF etiologies (6), including cardiomyopathies developed due to cardiomyocyte intrinsic defects or extrinsic pathologies and non-ischemic insults. Notably, mitochondrial defects comprise a hallmark of cardiomyocyte intrinsic abnormalities and are often linked to necrotic cell death, thus being an important target in HF (7, 8).

The mechanisms of cardiac pathophysiology and HF are conveniently studied in the mice with induced models of coronary ligation and aortic constriction, recapitulating, respectively, aspects of the initiation of ischemic and hypertensive cardiomyopathy and progression to HF. On the other hand, the availability of various genetic models of HF offers a plethora of additional perspectives, time points, and combinations of conditions for more proper investigation of cardiomyopathy and HF. Among them, the desmin-null mouse lacks one of the earliest expressing genes in cardiomyocytes and myocyte progenitors, which encodes the muscle-specific intermediate filament protein desmin [recently reviewed in (9, 10)]. Its importance for cardiac homeostasis has been clearly manifested by the development of mitochondrial defects, oxidative stress, myocardial remodeling, dilated cardiomyopathy, and HF in desmin-deficient mice (11–16). Actually, these mice combine features of multiple cardiomyopathies (12, 17, 18) and also serve as a model of cardiac injury with cardiomyocyte death, leading to a macrophage-driven inflammatory response, extended cardiac fibrosis and finally myocardial degeneration (17).

Both induced and genetic HF models readily manifest the disastrous effects of ineffective cardiomyocyte renewal and cardiac regeneration upon injury. As a hopeful exception to the above, the hearts of two widely used experimental animals, the zebrafish and the neonatal mouse (19–21), show a robust regenerative capacity and repair following an MI or other injuries. Studying the species-and developmental stage-specific differences that mechanistically support this success story may dictate novel routes of intervention in the failing human heart. In the present review, cardiomyocytes will be the main focus; however, cardiac fibroblasts and macrophages are increasingly recognized as major partners of cardiomyocytes and important regulators of cardiac homeostasis (22, 23). Therefore, understanding in detail the interactions of this “ménage a trois” may help us to decisively change their “rules of engagement” following myocardial damage. We enlighten here the most important points, focusing on mechanistic insights deduced from basic research and preclinical studies.

## Cardiac Subpopulations and the Contribution of Innate Immunity

### Cardiomyocytes and Their Progenitors

In the mouse embryo, the first differentiated myocardial cells appear in cardiac crescent at embryonic day E7.5. Two distinct cell lineages appearing by E8.5 segregate leading later to the formation of left and right ventricle, the atria and outflow tract. Myocardial sub-lineages have also been identified contributing to the arterial and venous poles [reviewed in (24)]. Adult mammalian myocardium consists of 30% cardiomyocytes (25) and ~1% cardiomyocyte progenitor or stem cells (CPCs, CSCs). Despite the lower estimations that occasionally appeared (26), cardiomyocytes occupy 90% of the adult heart's mass (27) and drive its principal function that is, to rhythmically contract and pumping blood to supply the body.

The majority of adult cardiomyocytes are quiescent, and although some studies suggest that they have a measurable capacity for turnover, this is very low (~1% per year) and insufficient to allow recovery to a functional myocardium, after a significant cellular loss (28–30). In the neonatal mouse, resident cardiomyocytes maintain a regenerative force and 1 day-old injured hearts can partially replace their parenchyma, an ability that will be soon lost, e.g., in animals older than 1 week (19). Recent studies showed that neonatal regeneration can also occur in pigs (31, 32). A similar situation in humans has been sporadically reported (33), conforming to a broader scar-less repair ability lost during development (34). This ability, however, is not limitless given that apical resection exceeding 15% of neonatal mouse tissue fails to regenerate (35). In addition, in other injuries such as MI it is debatable whether the neonatal mouse heart can successfully achieve scarless repair and regeneration (33, 36). Moreover, some groups reported absence of regeneration following resection (37). Recent studies in the mouse confirmed that there is, indeed, a time window allowing efficient postnatal cardiac regeneration but this is very short and the ability vanishes in 2 day-old animals (38). Similarly, neonatal swine hearts are able to regenerate following MI injury and this ability declines within 48 h (31, 32). Notably, fate mapping tools used in mice suggested that it is the resident cardiomyocyte populations and not CPCs or CSCs that give rise to the novel cells 3 weeks after an injury occurred at day 1 (19). Moreover, recent data based on the expression of a reliable marker for all cycling cells (ki67) and single cell transcriptomic analysis, revealed that a majority among cycling cells (about 10% of myocardial cells) in 1 day-old mice are cardiomyocytes and not CPCs or CSCs (39). The overall cycling activity drops to 0.05% in the adult murine heart and restarts reaching higher levels (about 1%) in an infarct after MI. However, in that case, the proliferating cells are mostly endothelial cells, fibroblasts, and macrophages, not cardiomyocytes. Importantly, ki67 lineage tracing followed up to 1.5 years in the same experimental settings failed to identify CPCs or CSCs among the sources of any cycling population, in contrast to a limited yet existing fraction of cycling cardiomyocytes (0.16% of cycling myocardial cells) emerged from pre-existing cardiomyocytes (39). Although previous studies suggested that during preadolescence a native cardiomyocyte number expansion occurs (40, 41), this is under debate (42, 43), and it is believed that the dramatic increase in mammalian cardiac size during this period is due to cardiomyocyte hypertrophy rather than proliferation (44).

An obvious question is whether there are intrinsic pathways that gradually block the ability of neonatal mammalian cardiomyocytes to proliferate. Which are the pivotal differences compared to cardiomyocytes of zebrafish, newts, and axolotls that retain this ability throughout lifespan? A particular cardiomyocyte characteristic that attracted attention is bi-nucleation, thought to be generated because of incomplete cytokinesis failing to capitalize persistent cardiomyocyte karyokinesis postnatally. In contrast to fetal heart that is comprised by mononuclear cardiomyocytes, the majority of the cardiomyocytes in the adult mouse are bi-nucleated (26) and the extent of bi-nucleation negatively correlates with cardiomyocyte's ability to proliferate and recover cardiac tissue following an injury (45). Accordingly, deletion of *Tnni3k*, a gene promoting bi-nucleation, enhanced adult mouse cardiomyocyte proliferation after injury (45). On the other hand, regenerating adult zebrafish hearts contain mononuclear diploid cardiomyocytes and when these were modified to achieve a degree of polyploidy either by *Tnni3k* overexpression (45) or *ect2* mosaic expression (46), their regenerative capacity was compromised. However, cardiomyocyte bi-nucleation represents a minor status in human hearts (29), thus failing to explain the lack of regeneration in our species. Moreover, in pigs, bi-nucleated cardiomyocytes increase from 10% at birth to only 30% in adulthood (47), again not explaining the switch of the regenerating neonatal heart. A possibly relevant, but not well-studied yet parameter, may be polyploidy (48), which is readily observed in adult swine and human hearts and to a much lesser extent in rodents, whereas zebrafish hearts contain only diploid cardiomyocytes.

On the other hand, the inability of cardiomyocytes to reenter the cell cycle has been linked to premature telomere dysfunction (49), nuclear interactions of the Hippo and Wnt signaling pathways (50), as well as to contribution of additional pathways including those of Notch (51) and neuregulin-ErbB (52, 53), albeit administration of neuregulin appeared inefficient in some settings (54). Forced overexpression of single or combinations of cell cycle regulators (cyclins and cyclin-dependent regulators) in mice had impressive beneficial effects in MI (55) and pressure overload [thoracic aortic banding (TAC) model] (56). However, in a setting of volume overload (aortocaval shunt), cyclin D forced expression failed to confer improved survival, cardiac function, and remodeling features (56). Nevertheless, there are obvious limits and risks in human therapeutic approaches when cell cycle reinforcing agents are used.

Moreover, cardiac regeneration and proliferation of cardiomyocytes may be regulated by their metabolic and oxidative status and hypoxia (57–59), as well as genes involved in mitochondrial quality control (60). Importantly, extrinsic cues such as physical interactions with extracellular space and matrix (61, 62) and even the innervation of the cardiac tissue (63) are crucial determinants. As discussed above, the native cardiomyocyte turnover in adult mammals, including humans (28, 64) is not enough to sustain cardiac integrity during injury, such as an MI, where millions of cardiomyocytes may be lost. As a consequence, replacement of myocytes by a fibrotic, non-contractile scar tissue occurs that might be initially helpful, but eventually compromises cardiac function, ultimately leading to HF (65). Even in the absence of injury, changes in the stiffness of the extracellular matrix surrounding the cardiomyocytes that occur during the first days of life, may impede the ability of cardiomyocytes to proliferate and consequently the capacity of the cardiac tissue to repair following an insult (38). Accordingly, cardiac stromal cells and macrophages, pivotal cellular determinants of the myocardial extracellular milieu, and their interactions with cardiomyocytes have lately attracted much attention as potential targets of intervention to improve cardiac repair.

### Cardiac Fibroblasts and Other Non-cardiomyocytes

Fibroblasts constitute a dynamic and versatile population of cells of mesenchymal origin that secrete collagen and other ECM components providing to neighboring cells a physical support to migrate, proliferate, differentiate, and properly function (23), thus being implicated in both regenerative processes and pathological conditions. Even though they have been commonly associated with disease, particularly through the development of fibrotic tissue, fibroblasts also produce mediators like growth factors, cytokines, and proteases and are involved not only in tissue homeostasis but also in repair and regeneration (23, 66, 67). Currently, there is no specific molecular signature able to accurately identify fibroblasts and since they exist in virtually any organ, they can express distinct phenotypic markers depending on their location (68). However, the combinatorial use of transgenic mouse lines expressing cell tracing markers under cardiac fibroblast specific promoters is a reliable manner of cardiac fibroblast tracking (69). Such markers include the ECM component Collagen1, the transcription factor Tcf21, the membrane receptor PDGFRα, as well as the matricellular periostin, the latter being expressed particularly by activated cardiac fibroblasts (70, 71).

In steady-state conditions, cardiac fibroblasts are responsible for maintaining proper ECM through a dynamic process of synthesis and remodeling, especially during the first week after birth. They contribute to modulation of physiological events, including the homogeneous distribution of mechanical stress and electrical insulation of the ventricles from the atria (19, 72, 73). On the other hand, by their biophysical interactions with cardiomyocytes through mechanical or electrical junctions, fibroblasts can facilitate electro-mechanical transduction important for the proper maintenance of the conduction system (74, 75). Upon insult, fibroblasts initiate production of ECM components for tissue reconstruction (76) and transmit signals to surrounding cardiomyocytes, but also to other stromal cells for initiation of the healing process (23, 77–79). However, alterations of fibroblast behavior in response to extrinsic factors, such as inflammatory cytokines, and persistent activation lead to deregulated matrix deposition and consequently to the formation of fibrotic tissue, underlining the pivotal role of fibroblasts in regulating the equilibrium between disease development and tissue repair and regeneration in the heart (70, 80–82) and other tissues (83–85).

Similarly to the stroma of other tissues (85, 86), myocardial fibroblasts are a heterogeneous population (87, 88). Most cardiac fibroblasts, including those of the annulus fibrosus and the leaflets of some valves, originate from the epicardium. Moreover, endocardium provides fibroblasts found in the atrioventricular junction of the septum, whereas a smaller population in the right atrium originates from neural crest. As highly specific markers and tools were lacking, estimations fluctuated during the last decades, but according to recent updates fibroblasts may account for <15% of the total myocardial cell population (25). This, however, changes both quantitatively and qualitatively upon injury.

In MI, fibroblast numbers increase during the proliferative phase. Fibroblast proliferation rates were previously reported to exceed that of endothelial cells following mouse MI injury (89). Within a newly formed infarct, proliferating fibroblasts peak 4–5 days after MI, whereas endothelial cells are dramatically reduced (69). Two to three weeks after MI the fibroblast proliferation rates return to basal levels, representing only 2% of proliferating myocardial cells. In other types of injury, expanding fibroblast populations occupy different locations, as for instance the arterial adventitia and perivascular areas in pressure overload and endocardial and septum areas after exposure to isoproterenol (simulating β-adrenergic cardiac stress). Nevertheless, they follow more or less similar expansion time courses (69).

Differentiation into myofibroblasts, a feature common to most fibroproliferative diseases (83), also occurs and is responsible for the production of high levels of collagen type I, and other cardiac ECM components. Cardiac myofibroblasts originate mostly from resident fibroblasts (87, 90) and may persist for months or longer, following MI or other injuries, whereas their ineffective removal has been correlated with the propagation of fibrosis and dysfunction in many organs including the heart (78, 83). However, following a cardiac insult, not all expanding fibroblasts are myofibroblasts (69, 87, 90). On the other hand, certain myofibroblast populations secrete MFG-E8 (milk fat globule epidermal growth factor 8) to remove dead cells from infarcted hearts, thus suppressing inflammation and restoring cardiac function (91). Accordingly, detrimental fibrosis *per se* (92), activated fibroblasts and associated molecules are being widely recognized as therapeutic targets in HF (81). In addition to the well-known TGF-β contribution to cardiac fibrosis (93), recent important advances include IL-11 that induces fibrosis acting downstream of TGF-β (94) and the small proline-rich protein 2b that induces proliferation of mouse and human cardiac fibroblasts (95). Additional specialized fibroblast populations have been identified in the infarcted heart that may sustain ECM changes and pathology (96), again by analogy to what happens in other tissues. Upon cardiac injury, fibroblasts may even adopt osteoblastic cell-like phenotypes, thus promoting detrimental cardiac muscle calcification (97). More recent transcriptomics data show that, at least in MI, cardiac fibroblasts sequentially assume different phenotypes which are important for cardiac repair (98). In particular, 1 day after injury fibroblasts become pro-inflammatory, they are highly proliferative and promote both fibrosis and angiogenesis 3 days later, whereas they inhibit angiogenesis after a week. Identification of molecular players regulating fibroblast polarization toward fibrotic or inflammatory phenotypes (99) is of obvious importance.

In recent years, an increasing amount of data has shown the great versatility of fibroblasts and the plasticity that characterizes them. A cardiogenic transcription profile and characteristics of cardiac mesenchymal stromal cells (MSCs), and pericytes has been revealed (100–103). Current engineering approaches try to take advantage of this plasticity and force their beneficial transformation into cardiomyocytes (see subsection on Engineering the fibroblast plasticity).

Apart from fibroblasts, in the complex cellular-extracellular milieu of the heart, additional non-myocytes forming part of the epicardial and endocardial layers and the vascular compartment interact with cardiomyocytes and fibroblasts and play important roles in cardiac repair and regeneration thus determining the final cardiac output in disease and HF. Due to space limitations however, we will only briefly describe selected regeneration-related aspects of vascular endothelial and smooth muscle cells, pericytes, epicardial and endocardial cells, and other less well-characterized populations [for specific reviews see (104–107)].

Recent estimations suggest that actually, endothelial cells comprise the most abundant myocardial population (25). Resident endothelial cells and not fibroblasts are the source of newly formed coronary vessels after injury (108), a process of great importance for regeneration (109, 110) and maintenance of heart function (104). Other vessel-associated cell types are important as well: Perivascular Gli1^+^ mesenchymal stem-like cells may differentiate into myofibroblasts upon aortic banding injury in the mouse, contributing to fibrosis (111) and pericytes that surround endothelial microvasculature are involved in perivascular inflammation and fibrosis promoting angiogenesis and regeneration (106). The outer epithelial layer (epicardium) serves as a major source of smooth muscle cells of coronary vasculature and cardiac fibroblasts in development and disease. Furthermore, it contributes to cardiac repair and regeneration by paracrinely inducing angiogenesis and cardiomyocyte division (112, 113) as demonstrated in zebrafish hearts (see respective section below). Endocardium, the inner cardiac endothelial layer, signals to the myocardium in development and endocardial Notch signaling activation may promote mammalian heart regeneration [reviewed in (114)]. Valve cells comprise distinct interstitial cell populations generated by endocardial cells through endothelial-to-mesenchymal transition (EMT), forming initially cardiac cushions at E9.5 and later the valves [reviewed in (24)]. Upon inflammatory signals, valve interstitial cells may respond in a manner similar to extra-cardiac fibroblast species leading to valve thickening and cardiac comorbidities, as in rheumatoid arthritis (115). Finally, telocytes comprise a fibroblast-like population with distinct morphological characteristics. Their position near potential cardiac stem cells and intense secretory identities may explain their anti-fibrotic and pro-regenerative effects which are used to improve heart pathology (116, 117).

Notably, many of the above cardiac populations share characteristics and markers with fibroblasts (118). Given the pronounced plasticity of the non-myocytes in cardiac development and disease, accurate definition of their molecular signature might be difficult to be achieved therefore many efforts focus on their beneficial manipulation.

### Cardiac Macrophages and Other Innate Immune Cells

Macrophages comprise 5–10% of total myocardial cells and are the most abundant leukocyte species in the heart (25, 119). Their identification is based on “classical” surface (F4/80) or intracellular (CD68) molecules, as well as several additional markers (CD11b, CCR2, Ly6C, MHC-II, MerTK, CD64, CX3CR1 being the most important in the mouse) used in various combinations in flow cytometry gating strategies or immunohistochemical studies. Some of these markers show various levels of expression (high, low) among macrophage subtypes and may be partially shared with other cardiac cells and immunocytes, including monocytes and dendritic cells. Recently CCR2 (the receptor of the MCP1/CCL2 chemokine) has been used to identify macrophages of monocyte origin.

During embryogenesis, macrophages firstly arise before definitive hematopoiesis, representing in the mouse at least four different subpopulations (120). The ontogeny of cardiac subpopulations includes primitive yolk-sac derived, fetal monocyte-derived, and adult monocyte-derived macrophages (121, 122). Among them, the yolk sac-derived CCR2^−^ macrophages populate the subepicardial space as early as E12.5 and are mostly located near the coronary vasculature, being also essential to its maturation (123). With the exception of the CCR2^+^ minor subpopulation, cardiac macrophages may follow monocyte-independent renewal in a homeostatic postnatal period. They maintain crucial functions, such as cardiac rhythm, by forming connexin 43-based gap junctions to electrically polarize cardiomyocytes in the cardiac conduction system (124). It was recently found that similar distinct subsets (CCR2^−^, CCR2^+^) constitute the macrophage compartment of the human heart (125), with CCR2^−^ macrophage renewal to be exclusively based on local proliferation, whereas the CCR2^−^ expressing species are monocyte-dependent. In cardiomyopathy patients, CCR2^−^ macrophages seem to locate near the coronary vasculature, similarly to what has been reported for mice, whereas CCR2^+^ macrophages occupy fibrotic areas (125).

Macrophages, in general, are highly plastic cells, adopting different, tissue- and milieu-dependent activation status (126). Their division into classically (M1) and alternatively (M2) activated, even if over-simplistic in some cases (127), has been helpful to describe macrophage populations that orchestrate inflammation (M1) or healing (M2), respectively (128, 129). A number of characteristic cell surface, cytoplasmic or nuclear markers were identified mostly from *in vitro* studies during the last years, whose presence or expression was considered to reflect M1 activation status or M2 and its variations (M2a, M2b, M2c). Accordingly, in a healthy and adult murine heart, the characteristics of resident macrophages would conform better to that of a polarized, alternative activation (130). However, timely emerging subtypes following a cardiac injury may be tissue-and case-sensitive, largely failing to be categorized according to the M1/M2 polarization paradigm (131), whose accuracy in representing the *in vivo* state is debatable (127, 132, 133). Recent pooled-cell transcriptomic analysis in the mouse MI model revealed that cardiac macrophages show high plasticity and assume diverse activation states, ranging from proinflammatory (resembling M1) to proreparative (resembling M2) within a short time window (7 days) after an injury. However, such states do not conform to the M1 and M2-states, in accordance to what is shown by independent single-cell transcriptomic analyses (134, 135), including the absence of the characteristic differential expression of iconic M1 or M2 markers, such as nitric oxide synthase 2 (*Nos2*) and arginase 1 (*Arg1*), respectively (136). However, it should be noted that gene expression and molecular marker distribution changes induced in cultured macrophages are indeed biased toward a “classical” or “alternative” activation state as described by M1 and M2 [also suggested to be replaced by terms denoting the polarization stimulus, e.g., M(IL4) and M(IFN-γ) (132)]. Although these may not coincide to the *in vivo* states, some markers and cytokines are still used to describe polarization and the paradigm has not been fully abandoned in the literature, including the characterization of cardiac pathology [e.g., (136, 137)]. Interestingly, macrophages can also mimic other cardiac cell types. Thus, the reparatory macrophages enriching an infarct region 1 week post-MI appear to overexpress collagen and periostin, both being acknowledged as cardiac fibroblast markers (136), underlining the need for detailed expression analysis of these cell populations of high plasticity at various time points following an injury, including longer-term conditions. What is happening, for instance, several months after an MI, where remaining fibrosis may sustain the course of the tissue toward heart failure?

During inflammation, such as after MI, inflammatory chemokines and cytokines released by the activated resident cardiac cells, including macrophages, stimulate bone marrow, and splenic reservoirs of stem cells and progenitors to produce in the blood and recruit in the cardiac tissue neutrophils and monocytes. These cells, in addition to digesting dead cells and removing debris, also release additional chemokines, cytokines, and enzymes sustaining inflammation. Distinct macrophage sub-populations may also emerge at early time points following an injury, such as MI, as for instance the blood-borne IFNIC (interferon-inducible cell) macrophage subset in which the type I interferon signaling pathway is activated (134). Actually, such cells may mediate detrimental MI inflammation. In the mouse, they sense as DAMP the DNA released by dying cardiomyocytes and respond through activation of the transcription factor IRF3 and associated type I interferon and interferon-stimulated gene expression in order to propagate inflammation.

Monocytes, on the other hand, originated from bone marrow, as well as splenic reservoirs (138), appear rapidly as Ly6C^hi^ (a surface marker) fraction and then, coinciding with neutrophil disappearance, a pro-repairing Ly6C^lo^ fraction, populating the post-MI heart (139, 140). Recently, a detailed analysis of the murine cardiac macrophage population, and the monocyte contribution to it in steady state and post-MI, was performed. This analysis was based on single-cell RNA-seq and the presence of known (MHCII and CCR2) and novel (LYVE1, a type I membrane glycoprotein binding hyaluronan, and TIMD4, a phosphatidylserine receptor) macrophage markers. Among the four macrophage populations identified as clusters with distinct expression profiles in the unbiased transcriptomic analysis, those expressing TIMD4 appear to be maintained independently of monocytes, whereas the rest are partially or fully replenished by monocytes (135). Notably, a monocyte population was also identified. Early after an infarct (day 2), resident cardiac macrophage levels are considerably reduced but they subsequently (days 4–28) slowly expand by proliferation. Importantly, in addition to the steady-state species, up to seven new myeloid cell subtypes, including four macrophage populations, can be identified following MI. TIMD4 expression appears to be a marker able to discriminate resident from CCR2^+^ monocyte-derived cardiac macrophages. Selective depletion of resident macrophages leads to exaggerated adverse remodeling, particularly in the peri-infarct zone. These results complement independent single-cell RNA-seq based studies described above (134) and suggest that in contrast to recruited macrophages, resident cardiac macrophages may actually exert a protective role in MI (135), at least in the particular time frames examined so far. The complete functional characterization of the multiple macrophage subtypes populating the mouse heart under steady-state conditions and injury (134, 135) will reveal the relative importance of each one of these populations as regards intervention targets in cardiac repair.

It is of interest that, among the similar sub-populations corresponding to the resident (CCR2^−^MHC^hi^) or recruited (CCR2^+^) species, also identifiable in human cardiomyopathy samples (135), the monocyte-dependent CCR2^+^ population shows proinflammatory features and its abundance is associated with persistent systolic dysfunction in patients undergoing LV-assisted device unloading (125). Moreover, the resident CCR2^+^ cardiac macrophages are responsible for the recruitment of monocytes in injured murine hearts, and, furthermore, they promote the differentiation of the recruited monocytes into inflammatory macrophage subsets. This occurs not only in MI but also following reperfusion injury or in a diphtheria toxin-based cardiomyocyte ablation setting (141). Another recent analysis identified three macrophage subpopulations dynamically expanding in somewhat distinct courses within the first 2 weeks after an MI (142). Among these populations, MHC-II^hi^/Ly6C^lo^ and MHC-II^lo^/Ly6C^lo^ expanding macrophages are of a proinflammatory phenotype (e.g., showing up-regulated expression of TNF family members), whereas Ly6C^hi^ macrophages assume a pro-reparative task by overexpressing genes promoting neoangiogenesis. Thus, distinct macrophage subsets in the mammalian heart respond to injury and differentially regulate repair processes following cardiomyocyte death-associated insults, suggesting that specific populations should be targeted in a time-dependent manner to achieve beneficial results.

As another example demonstrating a multifunctional role of macrophages and innate immunity, restriction of monocyte recruitment following viral myocarditis by IRAK-4 activation may actually lead to disease exacerbation exactly because interferon production will be suppressed and consequently viral survival and associated damage will be augmented (143). Reversely, mice deficient in interferon regulatory factor 3 or type I interferon receptor are protected from fatal MI. In that case, a reduced production of type I interferon by the DAMP-activated cardiac macrophages limits detrimental inflammation (134).

More changes may occur in cardiac macrophages following specific types of insult; over time, less IL-6, TNF, and MMP9 are produced in favor of higher levels of TGF-β and VEGF. This profibrotic and proangiogenic polarization, however, may have different impacts, as VEGF and angiogenesis appear to support regeneration, whereas a profibrotic response will harm the tissue in a longer term, leading to HF. Accordingly, in chronic rather than acute inflammatory injuries—such as TAC or angiotensin 2/aldosterone-induced pressure overload models in the mouse, roughly corresponding to HF with preserved ejection fraction (HFpEF) in humans—cardiac macrophage numbers are increased and mediate diastolic dysfunction by the production of profibrotic IL-10 (144). In particular, an overcrowding MHC-II^hi^ subpopulation seems to produce less MMPs and more osteopontin, thus promoting fibrosis. Similarly, we have found that osteopontin produced by macrophage-rich infiltrates in the desmin-null HF model, promotes over time cardiac fibrosis and dysfunction, mediated by an osteopontin-dependent galectin-3 secretion by macrophages (17). The temporally biphasic manner of macrophage contribution in the cardiac remodeling and potentially in the regeneration programs was shown in the TAC model of pressure overload, where macrophages initially proliferate and support angiogenesis in a KLF4-dependent manner (145). On the other hand, blockade of recruitment of macrophages that are linked to detrimental effects during late phase-pressure overload hypertrophy sustains cardiac angiogenesis and protects the heart from dysfunction (145). Alternatively, the expansion of CCR2^+^ macrophages in the left ventricle during the early compensatory phase following pressure overload in the mouse due to local up-regulation of CCR2 ligands is responsible for the subsequent CD4^+^ and CD8^+^ T-cell expansion in the mediastinal lymph nodes and the heart (146). These T-cell subsets actually mediate the late transition from hypertrophy to heart failure in this model (147, 148). Thus, early macrophage recruitment/expansion events may also trigger antigen presentation and T cell expansion leading to fibrosis and systolic dysfunction.

Changes in macrophage abundance and status occur also during aging (119, 144), sustaining a fibrotic cardiac phenotype. Aging-associated fibrosis, however, has also been attributed to events involving other cardiac populations such as accumulation of activated CD4^+^ T-cells (119) or differentiation of resident mesenchymal stem cells into fibroblasts that aberrantly secrete inflammatory cytokines, such as IL-6 and MCP-1 (82).

Additional cells of innate immunity regulate both the status and action of macrophages, as well as cardiac response to repair. Neutrophils, despite their short stay (e.g., up to 7 days in MI), affect macrophage polarization and promote cardiac post-MI repair, protecting from fibrosis and dysfunction (149). They can also adopt themselves different polarization states, analogous to those exemplified by macrophages (150). Therefore, their presence should not be considered anymore as plain pro-inflammatory but may have an important impact on remodeling and presumably on myocardial regeneration programs. The importance of macrophages in this interplay was underlined by recent findings showing that following cardiac injury the CCR2^−^ macrophages resident in the mouse heart promote the recruitment of neutrophils through TLR9/MyD88 signaling (151). Moreover, mast cells are also infiltrating the injured or stressed hearts and get activated to release enzyme and cytokines (for instance TNFα) important for adverse myocardial remodeling (152). Although a minor one, this innate immune cell population may both activate cardiac fibroblasts promoting detrimental fibrosis (153), and positively affect cardiomyocyte contractility following MI (154).

Another mononuclear phagocyte species, the antigen-presentation specialized dendritic cells (DCs), represents a minority among CD45^+^ cells in murine myocardium. Cardiac DCs are comprised by at least three subsets (135), among them two conventional DCs populations, cDC1, and cDC2 (155). In viral myocarditis, DAMPs secreted by dying infected cardiomyocytes lead to recruitment of phagocytes, such as monocytes and DCs. This allows for the generation of effector lymphocytes by viral antigen presentation of DCs populating the draining lymph nodes. CD103^+^ cDCs actually protect the murine heart from heart failure development following viral myocarditis, via cDC-mediated generation of antigen-specific CD8^+^ T-cells that promote viral clearance and resolution of cardiac inflammation (156). In steady state, cDC1s promote the development of regulatory T cell (T_reg_) (155), an immunosuppressive cell type of the adaptive immunity system, known to also restrict cardiac inflammation and MI fibrosis (157). During MI, DC infiltration and cDC2s activation induce the priming of autoreactive CD4^+^ T-cells specific to cardiac α-myosin that leads to the production of detrimental IFN-γ and IL-17 (155). Furthermore, it appears that depletion of cDCs from MI heart reduces IFN-γ and IL-1β expression and inflammatory infiltration of neutrophils and macrophages and improves cardiac fibrosis and function (158). Thus, despite earlier reports pointing out to a beneficial role of DCs in MI (159), a rather detrimental action of cDCs is currently described at least in the murine models of cardiac insult and repair examined so far. Further consequences of the adaptive immunity arm of DC activation in cardiac repair, however, are beyond the scope of the present review and the readers could refer to recent excellent reviews (22, 160).

Overall, macrophages and other innate immune cell populations undergo significant functional changes following cardiac injury and may exert both beneficial and detrimental actions in cardiac repair, in a case-specific and time-dependent manner. Accordingly, they have been the subject of intense research approaches aiming to precisely define their ontogeny, polarization, and contribution to cardiac remodeling and regeneration (22, 161–163).

**Figure 1:**
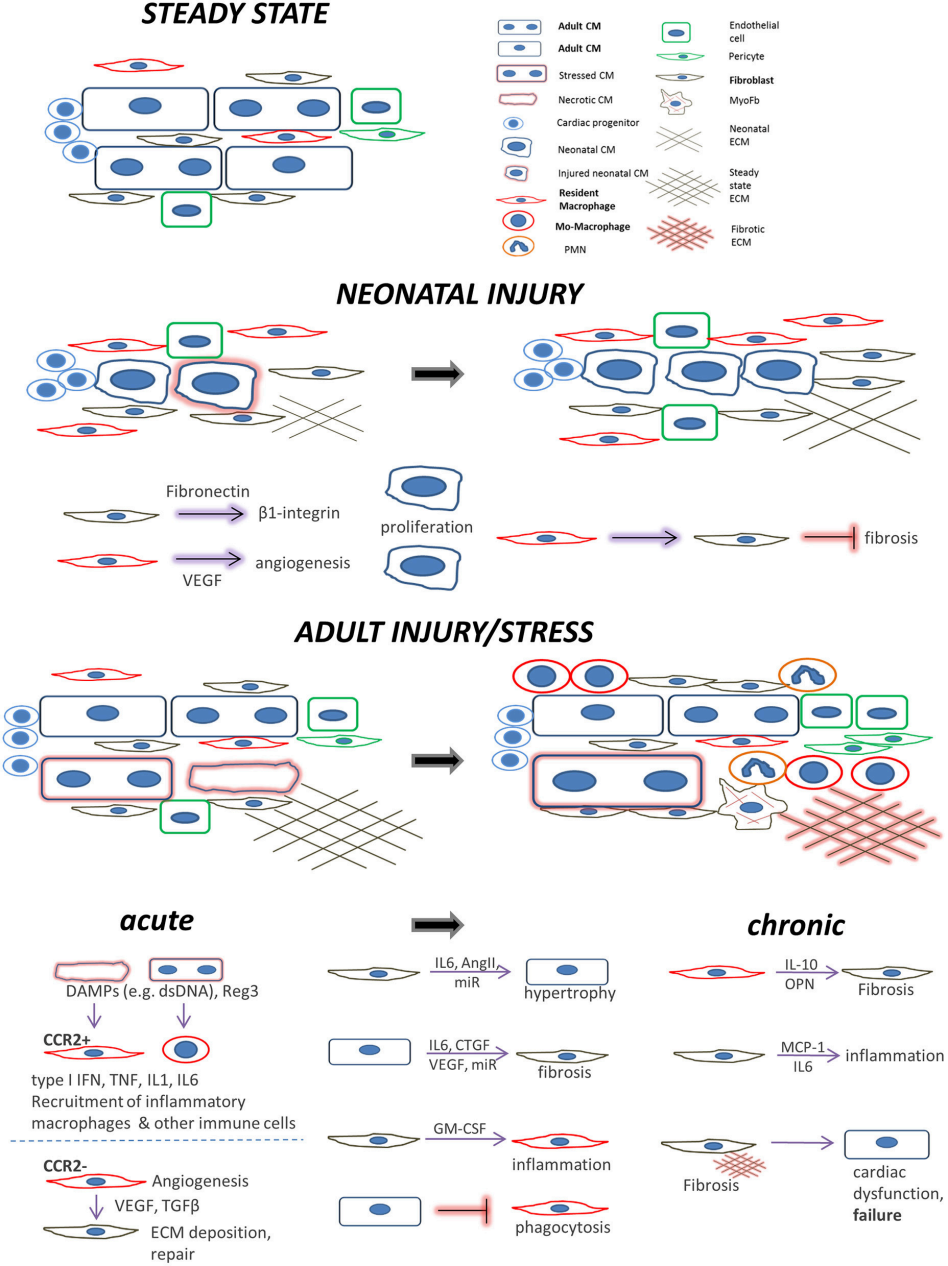
Main cell players during cardiac repair and regeneration: the interplay between cardiomyocytes, fibroblasts, and macrophages. *Healthy adult steady state*: Quiescent cardiomyocytes are in a homeostatic balance with interspersed cardiac fibroblasts, resident macrophages, and endothelial cells. *Neonatal injury*: cardiac regeneration happens only within a very short window postpartum. If injured, neonatal mammalian cardiomyocytes can proliferate in a permissive environment of appropriate stiffness. This is achieved with the help of (a) neonatal cardiac fibroblasts (which secrete factors to induce cardiomyocyte proliferation via integrin signaling) and (b), resident, yolk-sac derived, CCR2^−^ macrophages which secrete proangiogenic factors or instruct fibroblasts to assume alternative, less fibrogenic states following an injury. *Adult injury*: almost no regeneration occurs in the adult myocardium. Under *acute injury* conditions (myocardial infarction, infection, intrinsic cardiomyocyte defects, etc.) dying cardiomyocytes release danger signals (DAMPs) leading to mobilization and recruitment of monocytes and generation of monocyte-derived macrophages. Additional cardiomyocyte derived mediators such as the Reg3 proteins contribute to inflammatory macrophage expansion. CCR2^+^ resident macrophages recruit more monocyte-derived inflammatory macrophages and secrete agents (e.g., type I IFN, IL-1, TNF) propagating inflammation, whereas they can also recruit detrimental T-cells (not shown). On the other hand, CCR2^−^ resident macrophages and macrophage subsets emerging at later time points following injury, assume a pro-reparatory phenotype by expressing and releasing pro-fibrotic and proangiogenic factors, mainly through activation of fibroblasts and ECM deposition, to promote cardiac repair, and also by recruiting pro-reparative neutrophils (PMN). In addition, macrophage phagocytosis action promotes cardiac repair and is probably prevented by cardiomyocyte signals. Moreover, fibroblasts interact with cardiomyocytes in a paracrine manner to promote hypertrophy and fibrosis and instruct macrophages to mediate inflammation by secreting agents such as GM-CSF. *Chronic stress and injury:* macrophage activation in early time points affects the outcome. The inflammatory macrophage expansion in early pressure overload injury, for instance, leads to fibrotic ECM and heart failure in later time points triggering detrimental T-cell expansion (not shown). Under chronic activation conditions (pressure overload, cardiomyopathies, aging) macrophages secrete factors promoting fibrosis (e.g., IL-10, osteopontin) and fibrosis mediated by activated fibroblasts (including myofibroblasts) stimulates additional pathways affecting cardiomyocyte integrity and function. This propagates the detrimental effects of inefficient regeneration and repair, leading to heart failure. Fibroblasts can also mediate inflammation by releasing inflammatory cytokines such as MCP1 and IL6.

The macrophages, in particular, being the major cardiac innate immune population, may exert detrimental excessive inflammation at early time points after an acute injury mediated by its monocyte-derived arm, whereas the resident macrophage subset mediates rather cardioprotective actions. Their subsequently emerging reparatory phenotypes further induce cardiac repair but can be also detrimental in the longer term by sustaining fibrosis, particularly under chronic stress conditions or aging (Figure 1).

Particular characteristics of neonatal macrophages may decisively contribute to the maintenance of cardiomyocyte regenerative capacity in a mammalian neonatal heart. In the pro-regenerative environment of the heart during the first week of postnatal life the yolk sac-derived CCR2^−^ macrophages expand by proliferation, whereas the newcomer CCR2^−^ population of hematopoietic origin enters the regeneration-hostile heart by the 2nd week of life (120, 122). Deletion of CCR2^−^ derived macrophages from the neonatal murine heart blocks its transient regenerative response to MI, leading to fibrotic scar and regenerating hearts of 1 day old mice are enriched in macrophages in contrast to the non-regenerating fibrotic hearts of infarcted 14 day-old mice (164). Further analysis revealed that although cardiomyocyte proliferation was not directly affected in the macrophage-depleted infarcted hearts, angiogenesis was severely compromised. In line with the higher expression of pro-angiogenic genes observed in macrophages from regenerating hearts, it was deduced from these data that neonatal cardiac macrophages support neoangiogenesis instead of fibrosis, thus promoting cardiac regeneration (164).

We will next summarize the contribution of macrophage interactions with cardiomyocytes and cardiac fibroblasts in the regulation of cardiac injury, regeneration, and repair (Figure 1), including attempts to beneficially manipulate these interactions to decrease the negative consequences of myocardial injury.

## Cardiomyocyte-Cardiac Fibroblast-Macrophage Interactions

Cardiomyocytes and interspersed interstitial cells such as fibroblasts are adjacently situated in the heart and can interact via the release of paracrine factors, direct cell-cell interactions and indirectly through ECM (165). Cardiomyocytes and interstitial cells, including resident macrophages, possess specialized structures to interconnect and communicate (77, 124). Gap junctions (formed mostly by connexin 43 and 40) and adherens junctions mediate electrical and mechanical conduction and are important for the development of arrhythmias and fibrosis in a diseased heart. In addition, long thin membrane nanotubular structures have been observed between cardiomyocytes and fibroblasts *in vitro* and *in vivo* (166), and it was suggested that they might allow mitochondria exchange and calcium signal propagation. We will mainly focus on the interactions mediated in a paracrine manner among these three cell populations and their consequences for cardiac repair and regeneration (Figure 1).

Several *in vitro* approaches using co-cultures or exposure of one cardiac cell type to the secretome of the other have revealed effects of communication and interaction primarily between cardiomyocytes and fibroblasts. Some of their conclusions were supported by or translated to successful preclinical interventions.

Thus, in a paracrine fashion, fibroblasts may trigger cardiomyocyte hypertrophy via secreted angiotensin II or microRNA species, whereas cardiomyocytes can induce fibroblast proliferation by secreting IL-6 (167, 168). In contrast to adult fibroblasts that induce hypertrophy, embryonic fibroblast-derived signals promote cardiomyocyte DNA synthesis through β1-integrin signaling (169). Similarly, macrophages derived from neonatal -but not adult- mouse hearts secrete angiogenic factors such as VEGF and instruct cardiomyocytes to proliferate (164). Vice versa, in hypertrophic cardiomyopathy, mutations in sarcomeric proteins in cardiomyocytes can lead to increased profibrotic gene expression and proliferation of non-myocytes (170). This vicious cell interaction circle was recently confirmed by demonstrating that activation of p38α in adult cardiac fibroblasts, under β-adrenergic receptor stimulation, leads to IL-6 secretion that paracrinely causes cardiomyocyte hypertrophy (171). On the other hand, stimulation of protein kinase A in cardiomyocytes leads to CTGF and VEGF secretion that paracrinely induce collagen production and fibroblast proliferation leading to fibrosis (172).

Apart from hypertrophic signaling, cardiomyocytes may also secrete molecules, such as micro-RNAs to paracrinely stimulate collagen production via activation of the TGF-β1 pathway in co-cultured cardiac fibroblasts (173). However, the relative contribution of TGF-β1 pathway activation to fibrosis differs among the two cardiac populations. Whereas, cardiomyocyte-specific deletion of Smad3, a pivotal downstream effector in the TGF-β1 pathway, protects them from apoptosis in MI and restricts adverse remodeling, fibroblast-specific deletion leads to their hyperproliferation with a concomitant reduction of collagen synthesis leading to post-MI cardiac rupture (174).

Upon cardiac injury, factors such as GM-CSF secreted by activated fibroblasts can instruct resident macrophages to recruit inflammatory monocytes and neutrophils and propagate MI inflammation (175). Similarly, in a mouse autoimmune myocarditis model simulating inflammatory dilated cardiomyopathy, IL-17A, an inflammatory cytokine, induces GM-CSF production by cardiac fibroblasts, which in turn paracrinely instruct proinflammatory differentiation of monocytes *in vitro* and cardiac infiltration of Ly6C^hi^ monocytes/macrophages (176). Interestingly, cardiac infiltrating Ly6C^hi^ macrophages mediated pathology in this case by overexpressing both proinflammatory and profibrotic genes. On the other hand, the activation status of macrophages appears to play a role in cardiac fibroblast behavior. Accordingly, infusion of anti-inflammatory IL-10 into infarcted hearts conferred alternative type polarization to cardiac macrophages, which in turn stimulated fibroblast proliferation and collagen production, thus protecting against MI damage in the short term (177). Moreover, MI in mice lacking TRIB1 -a member of the Ca^+2^/calmodulin-dependent protein kinase (CAMK) of the Ser/Thr protein kinase family- that show selective depletion of alternatively activated macrophages led to the suppression of fibroblast activation, reduced collagen deposition and fatal cardiac rupture. These events were reversed by IL-4 or re-administration of alternatively activated macrophages themselves, which seem to beneficially activate fibroblasts in this setting by secreting osteopontin and IL-1α (178). However, as we noted above, macrophage expansion may be detrimental in other time points and types of injury as occurs for instance under conditions of pressure overload or in the desmin-null mouse. Another important aspect of macrophage-fibroblast interaction in the heart comes from studying cardiac regeneration in another model than zebrafish, the urodele amphibian salamander. In this regenerating cardiac milieu, macrophage depletion results in a permanent and highly cross-linked fibrotic scar and cease of regeneration (179). This, however, is not directly owing to affected cardiomyocyte proliferation and is rather a result of macrophage-mediated altered fibroblast proliferation counteracting efficient cardiomyocyte proliferation *per se*.

Cardiomyocyte-macrophage interactions are also important for cardiac repair [recently reviewed in (180)]. As already described, cardiomyocytes releasing DAMPs under stress or injury recruit inflammatory monocytes and monocyte-derived macrophages (134). Inhibition of the responsive pathways by deletion of pattern recognition receptors (PRR) in mice (e.g., the cyclic GMP-AMP synthase—a stimulator of interferon gene pathway) results in polarization of macrophages toward a reparative phenotype and protects against MI adverse remodeling and rupture (181).

The release of DAMPs is a characteristic of necrotic cell death and pyroptosis (182). In that case, activation of RIPK1 and RIPK3 protein kinases and necrosome formation (183) or inflammasome formation and caspase-1 activation (184) lead to loss of plasma membrane integrity and cell lysis, releasing DAMPs that mediate inflammation upon tissue injury, including cardiac injury (3, 185, 186). In contrast, apoptotic cell death, mediated either by caspase 3, 6, or 7 activation via the cell death receptor (extrinsic) or mitochondrial (intrinsic) pathways, maintains membrane integrity but leads to the release of “eat me” signals to trigger phagocytosis of apoptotic cells (182). These events (called efferocytosis) actually activate the generation of anti-inflammatory signals, such as the production of TGF-β and IL-10, by the phagocytosing macrophages (187), to terminate inflammation. Macrophage efferocytosis of apoptotic cardiomyocytes is regulated by both myeloid-epithelial-reproductive tyrosine kinase (MerTK) and MFG-E8 and when compromised, the acute inflammation following for instance an MI fails to resolve, leading to enhanced adverse remodeling (188, 189). Moreover, resident reparatory CCR2^−^ macrophages express high levels of MerTK and release TGF-β upon cardiomyocyte engulfment (190). On the other hand, dying cardiomyocytes in MI may hinder their phagocytic removal by inducing shedding of macrophage MerTK (191). Both apoptosis and necrosis characterize cardiac cell death in MI and heart failure (192, 193), differentially contributing to pathophysiology. These forms of cell death may prevail at different time points (194) and cell populations (195, 196), with apoptosis being mostly responsible for non-myocyte death, including macrophages themselves. Nonetheless, cardiomyocyte apoptosis, even if limited, is considered important for the long term development of heart failure pathophysiology, given the inability of the cardiomyocyte population to regenerate.

Apart from such interactions, cardiomyocytes can also more directly instruct macrophage polarization and activities. Thus, extracellular vesicles isolated from mouse neonatal cardiomyocytes, when applied on macrophages induce p38MAPK activation and inflammatory gene expression (197). Moreover, early after MI, cardiomyocytes transiently overexpress and release several Reg3 proteins, members of the C-type lectin family (142). Local administration of Reg3 protein paralogues in mice promoted recruitment of pro-inflammatory macrophage subsets (MHC-II^hi^/Ly6C^lo^ and MHC-II^lo^/Ly6C^lo^), whereas the pro-angiogenic and reparative Ly6C^hi^ subpopulation was suppressed. Moreover, under ischemic conditions, cardiomyocytes seem to induce via secreted vesicles the adhesion of macrophages to ECM and lead to the reduction of their ability to phagocytose, suggesting a paracrine cardiomyocyte-macrophage crosstalk (197) that impairs debris clearance in MI. The debridement of damaged cardiac tissue is an important parameter, as its impairment by depletion of macrophages following cryo-injury in the mouse leads to diminished myofibroblast infiltration and vascularization and a thinner free wall accompanied by increased dilatation and mortality rate (198).

Most of our current knowledge on paracrine cardiac cell interactions stems from conventional two dimensional (2D) cell culture experimentation, mainly analyzing the effects caused by co-culturing two distinct isolated cardiac populations or exposure of one cell type to the secretome of another. Although informative, such approaches ignore important parameters such as specific topological arrangements in a healthy or diseased heart. Thus, in a fibrotic heart, fibroblasts may form aggregates at certain time points. Mimicking this condition *in vitro*, by spherical rearrangement of cardiac fibroblasts from a 2D monolayer to a 3D cluster, revealed that fibroblast expression profiles and secretory activity better conform to those prevailing in a remodeling heart (199). These 3D culture signatures are characterized by reduced proliferation, ECM and contractile protein synthesis and increased ECM degradation activity (with a pronounced up-regulation of MMP-11), chemotaxis and inflammation. Importantly, conditioned media from the 3D fibroblast cultures induce a stronger hypertrophic response of neonatal cardiomyocytes than the 2D counterparts (199). Moreover, 3D spheroids of mixed populations (e.g., cardiomyocytes and fibroblasts) with the use of directed fusion allow the build-up of heterocellular microtissues recapitulating the spatial distribution of cardiac cells and provide new tools to study parameters of arrhythmias and fibrosis (200). Going a step forward, scaffold-based 3D cultures include ECM components and may be game changers in understanding the real *in vivo* relations and effects under altered stiffness conditions, as those occurring for instance in a fibrotic MI scar. These so-called cardiovascular organs-on-a-chip approaches (201, 202), also serve as tools to understand the molecular basis of cardiac diseases and test drug efficiency, approaching the precision medicine level. Moreover, heart slices may represent a faithful tissue replica (77, 165).

Many of the known effects of cardiomyocytes on cardiac fibroblasts and vice versa should be re-examined using similar novel approaches to achieve evaluations a step closer to the *in vivo* state. Importantly, the introduction of resident macrophages in these systems will greatly precipitate our understanding of cardiac repair and regeneration mechanisms.

## Exploitation of Cardiac and Modified Extracardiac Cells and Their Interactions to Orchestrate Cardiac Repair

The pronounced inability of the adult human heart to regenerate motivated numerous interventional approaches, mostly based on previous mechanistic studies in mouse and zebrafish models (4, 105, 118). These efforts targeted the cardiomyocytes themselves, their potential progenitors, extracardiac stem cells, cardiac fibroblasts, and immune components. Despite acknowledged limits and some disappointments (203), a vast amount of knowledge was gained pointing out immune response manipulation as well as engineering of fibroblasts, stem cells and their tissue environment as the most promising therapeutic targets, some already being the subject of ongoing clinical trials (204). In the following four subsections, we briefly summarize the main goals and achievements of such approaches, particularly focusing on the importance of the interactions among the main cell players and their exploitation to orchestrate cardiac repair.

### Lessons From the Zebrafish Models

Zebrafish is a unique vertebrate animal model that combines the ability to regenerate its organs throughout its life, as well as allowing non-invasive *in vivo* imaging and genetic manipulations (205). Tail fin amputation (206), central neural system injury (207), retina (208) and heart injury models have been developed to explore the remarkable regenerative potential of zebrafish. In particular, concerning the heart, several injury models have been established: resection (20), cryoinjury (21, 209, 210), and chemogenetic ablation (211, 212). The latter refers to the use of metronidazole/nitroreductase combination or the tamoxifen-inducible tissue-specific expression of Diphtheria toxin A. Both approaches allow inducible tissue-specific damage and offer the possibility to study regeneration in different organs and developmental stages that are not surgically amenable. This approach allowed following *in vivo* the regeneration of pancreatic beta cells (211, 213), cardiomyocytes, hepatocytes, macrophages, podocytes, retinal neurons subtypes, etc. [reviewed in (214)]. These studies revealed that the reactivation of developmental pathways seems to be a recurrent theme during regeneration, as well as that an early stage acute inflammatory phase is beneficial for the regenerative response.

Inflammation is required and sufficient for enhancing the proliferation of both neural progenitors (207) and zebrafish cardiomyocytes (215). Medaka (Oryzia latipes) failed to activate a robust inflammatory response and recruit enough macrophages, upon cardiac cryoinjury, and subsequently showed impaired regeneration potential (216). In this context, activating Toll-like receptor signaling enhanced the immune response and neutrophil clearance, promoting regeneration also in medaka. An inflammatory response leads to fibrosis, but zebrafish provides a paradigm where fibrosis regresses, implying that fibrosis, and regeneration after cardiac injury are not mutually exclusive. Zebrafish resected hearts form a fibrin clot, or myocardial cells undergo necrosis after application of a liquid nitrogen-cooled probe, and collagen scars or fibrin deposits are primarily formed. However, and in contrast to mammals, they are subsequently replaced by new cardiomyocytes within 60–90 days (20). Upon cryoinjury and cardiomyocyte damage, leukocytes are attracted to and populate the damaged area. ECM proteins accumulate at the injury site, and a transient fibrotic tissue is formed. However, in contrast to the situation in mammals, fibrosis regresses and eventually is resolved. Fibroblasts remain at the damaged area but are inactivated, shutting down their profibrotic program (217). Identifying, therefore, the signaling pathways that drive fibroblast inactivation in zebrafish to enhance the regenerative potential of mammals is an intriguing hypothesis.

Three major regeneration events occur in the zebrafish heart following damage. The epicardium is activated by re-expressing embryonic markers and contributes to new vascularization of the regenerating myocardium. Cardiomyocytes start to proliferate to replace lost myocardium. The transient fibrotic tissue regresses. The signaling molecules guiding this regenerative response in fish remain largely unknown. *In vivo* high-throughput chemical screens of a fluorescent cell-cycle indicator transgenic line already identified several molecules that could induce cardiomyocyte proliferation at the early embryonic stages. These molecules have been shown to also facilitate heart regeneration in adult zebrafish (218). The endocardium and epicardium are activated to produce retinoic acid that supports cardiomyocyte proliferation as a paracrine factor (219). The epicardium is a key tissue for regeneration that secretes several essential factors for the proliferation and survival of cardiomyocytes. Additional signaling pathways identified to be important for heart regeneration are the Hedgehog (220), Notch (221), Sdf1a (222), Pdgf (223), and Vegfaa (224). However, *Vegfaa* exhibited a dual role in the context of cardiac injury enabling ectopic cardiomyogenesis but inhibiting regeneration at the site of the injury. Recently published studies of the Mexican cavefishes that fail to regenerate their hearts when compared to their surface fish relatives unraveled several quantitative trait loci that are linked to this ability. The leucine-rich repeat containing 10 gene (*lrrc10)* was already verified as necessary for proper heart regeneration whereas several more candidates may prove to be pivotal (225).

Preconditioning zebrafish hearts by several means, including remote stimulation by peritoneal sterile inflammation, also induces cardiomyocyte re-entry into the cell-cycle and boosts their regeneration potential (226). Inflammation is thought of as a double-edged sword having both detrimental and beneficial consequences. It is becoming therefore evident that instructive factors for heart regeneration, as well as inflammation, require tight spatiotemporal control for efficacy. A major challenge becomes to regulate this spatiotemporal response and identify the beneficial signals to promote regeneration while maintaining the ability to resolve fibrosis.

### Using Cardiac Progenitors and Induced Progenitor Cells

Endogenous cardiac progenitor cell (CPC) populations that reside at small clusters in niches within the postnatal myocardium raised new possibilities for cardiac regeneration. Despite recent evidence suggesting that most probably there is no true stem cell in the human and experimental rodent heart and that any newly formed cardiomyocytes in steady state or after injury are actually the descendants of pre-existing mature cardiomyocytes (39, 227), mobilization or administration of native or *ex vivo* modified CPCs or their products has been proven beneficial in preclinical protocols and deserve further exploitation. A variety of resident CPCs have been identified in the adult mammalian heart according to their expression of the cell surface markers c-kit (228) and Sca-1(229), the transcription factor Islet-1 (230) or according to their ability to form Cardiospheres (CS) *in vitro* (231). All the above were shown to have proliferative properties and differentiation capacity toward cardiomyocytes, endothelial and smooth muscle cells. A novel population expressing the transcription factor twist-2 was recently demonstrated to contribute to cardiomyocytes, endothelial cells and fibroblasts in the adult murine heart as well (232). Resident cardiac Side Population (CSP) cells that are defined by their ability to efflux the dye Hoechst33342 from the cytoplasm (233, 234) are another source of progenitor cells for heart repair, as they can differentiate toward all cardiac lineages (235–238). Both CSP and CS-derived cells represent more heterogeneous populations (231, 239), expressing mostly the markers Sca-1 and c-kit, respectively, among others, while Islet-1^+^ cells represent remnants of an embryonic progenitor population that contributes to the formation of the second heart field during development (240). A significant contribution to cardiac regeneration may also arise from epicardium, as priming with thymosin β4 before injury resulted in re-expression of the key embryonic marker Wt1 in epicardial progenitor cells, causing them to invade the myocardium and to differentiate into cardiomyocytes (241). Notably, c-kit^+^ (242) and CS-derived cells (243) have undergone clinical trials to treat ischemic myocardium. Despite the reduction of the infarct size, the overall improvement of cardiac function was moderate with minimal engraftment of the cells within the myocardium, thus entering their cardiomyogenic potential under debate (244–246). Indeed, recent genetic lineage tracing approaches for the c-kit and Sca-1 markers showed that these CPCs do not contribute significantly to new cardiomyocyte formation during development, with aging or after acute injury, in contrast to what was believed before, but rather differentiate to vascular lineages (244–247).

Although c-kit^+^ and Sca-1^+^ CPCs are not considered anymore as a native source of cardiomyocyte producing progenitors, nonetheless they augmented the function of injured heart upon transplantation, which was also reported for CSP and CS-derived cells in animal models, likely through a paracrine manner (248–252). The latter implies that the regenerative potential of the heart is not only determined by the characteristics of CPC but is also influenced by the various cellular interactions and the microenvironment. Indeed, a great body of studies indicate that the constitution of the ECM, secreted molecules such as pro-inflammatory cytokines and chemokines, mechanical forces, the cellular interactions of CPCs with the stromal cells or the immune components of the myocardium, as well as the disease conditions that possibly predominate the cardiac tissue, may impact the proliferation, aging and differentiation capacity of CPCs and, consequently, positively or negatively regulate heart regeneration (61, 249, 253–258) [also reviewed by (259)].

Interestingly, immune cells seem to play a profound role in muscle regeneration either by regulating cardiac remodeling or/and cardiomyocyte proliferation (164, 260). Moreover, an intact immune response is essential to achieve any improvement observed in cardiac function when injecting bone marrow derived progenitor cells into infarcted myocardium (261). Furthermore, bone marrow-derived MSCs achieve better improvement in cardiac function, fibrosis, and angiogenesis when administered in combination with co-cultured macrophages in rat MI, the improvements being associated with elevated anti-inflammatory macrophages and gene expression (262). Finally, the inflammatory response *per se* elicited during and owing to a stem cell-based therapy may be of paramount importance for the beneficial effects of the latter in a diseased heart (263). Indeed, the amelioration of cardiac dysfunction observed when c-kit^+^ CPCs or fractionated bone marrow mononuclear cells are injected in mouse hearts undergoing ischemia/reperfusion is not owing to newly generated cardiomyocytes, but rather to the associated inflammatory response that leads to improved mechanical properties of the infarcted area (264). In particular, direct injection of these cells in the heart triggers a local accumulation of CCR2^+^ and CX3CR1^+^ macrophages that alter fibroblast activity and ECM content in the border zone of the infarct improving cardiac output. Notably, similar improvements can be achieved by the injection of non-cellular activators of innate immune response such as zymosan (264), further underlining the pivotal role of inflammation in cardiac fate after an injury.

Serving as an important alternative to CPCs, the induced Pluripotent Stem Cells (iPSCs) are extracardiac stem cells that originate from the reprogramming of somatic cells via the ectopic expression of the transcription factors Oct3/4, Sox2, Klf4, and c-Myc and have wide differentiation ability, giving rise to all cell types of the three germ layers (265). Their cardiovascular differentiation capacity has been studied *in vitro* and *in vivo* with beneficial results regarding cardiac function after MI in mice (266–269). The use of human iPSCs for myocardial regeneration circumvents the low available number of resident CPCs, as well as suboptimal cell engraftment upon transplantation, two additional hurdles with CPC therapy. However, the disadvantage of teratoma formation by iPSC still persists (270, 271). iPSC-derived cardiomyocytes (iPSC-CMs), representing a source of differentiated/mature cells, overcome this limitation and their use to heal the diseased myocardium of small and primate animal models showed improvement of cardiac function after transplantation (272–274). Moreover, human iPSC-CMs can serve as an alternative to animals for modeling human diseases, drug testing, and development of personalized therapies, since animal models often do not accurately reproduce human pathophysiology. Over the past years, more than 70 human diseases and cardiomyopathies have been modeled using iPSC-derived cells, including cardiac sodium channel diseases, the long QT syndrome, a desmin-related dilated cardiomyopathy, Barth syndrome and Duchenne muscular dystrophy (201, 275–277).

Future cardiac regeneration efforts are focused on taking advantage of gaining knowledge from trial and error so far, in combination with technological advances. Toward this direction, a lot of work is done on increasing CPC and iPSC-CM engraftment and survival after transplantation to the heart using scaffold-based approaches, such as hydrogels or biomaterial cardiac patches on which cells are seeded and expanded *in vitro* prior to transplantation, or scaffold-free cell sheets (278–281). Importantly, these approaches take into account the spatial interactions among cardiac cells and their surrounding matrix that are of most importance in the complex myocardial tissue, as also repeatedly underlined in the previous sections. Introduction of CPCs and iPSC-CMs after injury via these methods resulted in increased cell survival, long term retention to the host myocardium and increased cardiac function (278, 279). The advantage of a patch-based approach over intra-myocardial cell injection for cardiac repair may rely on simultaneously acting as a substrate that strengthens the injured myocardium and prevents adverse remodeling, and as a template for cells to survive and proliferate. In addition, the use of stem cell-derived exosomes, as a cell-free alternative for stem cell-based regenerative approach, induced heart regeneration and augmented cardiac function when administrated after injury (281, 282). Their beneficial effect relies on their composition of soluble factors and macromolecules, mostly specific microRNAs that are transferred by extracellular vesicles to cardiomyocytes (282–284).

It is important to note that the integration of cardiac cell-cell and cell-ECM interactions in all these approaches is crucial. Accordingly, the particular cell and ECM composition and interactions in the pro-regenerative milieu of rodent neonatal hearts decisively promote the maturation of mouse stem cell-CM and human iPC-CM preparations toward adult cardiomyocytes (285). Moreover, mouse embryonic stem cell-derived CMs cultured in microtissues and exposed to conditioned medium from fibroblasts obtained from pro-regenerative neonatal hearts, assumed better spreading and contractile activity compared to those exposed to medium from adult fibroblasts (286). Thus, cardiac cell interactions from a pro-regenerative environment can be exploited to induce maturation of engineered cardiomyocytes, expected to allow better regeneration properties of future intervention protocols. Additional aspects regarding the particular contribution of innate immune cells are discussed under the Manipulating the Immune Response section underneath.

### Engineering the Fibroblast Plasticity: Transdifferentiation to Cardiomyocytes

Fibroblasts have been extensively used in order to uncover new potential applications in regenerative medicine. Despite their increased use as a source of iPSCs the immunological side effects, the oncogenic potential of the remaining undifferentiated cells and the low engraftment of transplanted cells, potential hurdles of iPSC technology application, urgently called for an alternative approach.

These challenges can be bypassed by the direct reprogramming of differentiated somatic cells into a different, distinct somatic fate without passing first through a pluripotent state. Indeed, an alternative to iPSC protocol using the ectopic expression of a combination of three genes encoding the cardiac transcription factors, *Gata4, Mef2c*, and *Tbx5* (GMT), successfully induced reprogramming of murine fibroblasts into cardiomyocyte-like (iCM) cells *in vitro* (287). This promising achievement proved later to be also transferrable *in vivo* [reviewed in (4)], thus constituting an alternative route to support cardiac regeneration using an abundant cardiac cell population.

iCMs express major cardiac genes and exhibit cardiomyocyte characteristics, such as sarcomere structure, spontaneous intracellular calcium oscillations, and beating contractions (287). Other studies showed that the addition of other transcription factors, such as HAND2 (GHMT cocktail) (288), transcription repressor molecules (ZNF281) (289), miRNAs, and growth factors can enhance the reprogramming efficiency (290–294). Suppression of certain genes encoding factors such as the Polycomb complex protein BMI1 and the splicing factor polypyrimidine tract-binding protein 1 (PTB) (88, 295) or manipulation of major signaling pathways such as those of Wnt, Notch, TGF-β, and the serine/threonine-protein kinase, protein Kinase B (AKT1) pathway also enhanced cardiac reprogramming (296–299). In addition to direct reprogramming, there has been partial reprogramming of fibroblasts into CPCs by using a cocktail of five genes encoding early cardiac factors -Mesp1, Gata4, Tbx5, Nkx2-5, and Baf60c (300). These progenitors are an expandable multipotent -and not a pluripotent- cell population, aiming to increase in numbers the iCMs, and be able to replace the lost cardiomyocytes after insult (300). In both cases of direct and partial cardiac reprogramming, transplantation of reprogrammed fibroblasts into immunodeficient murine hearts after MI, resulted into (i) differentiated cardiomyocytes, in actually higher numbers than in previous *in vitro* studies, suggesting also a paracrine favorable factor, (ii) endothelial cells, and (iii) smooth muscle cells, as well as improved survival (287, 300). The next step in *in vitro* direct reprogramming is the use of human fibroblasts to generate functional iCMs. Whereas this was proven to be more challenging, it was achieved with the addition to the original GMT or GHMT viral cocktail of factors such as myocardin (MYOCD), MESP1, estrogen-related receptor-γ (ESRRγ), and zinc-finger protein ZFPM2, or miR-1 and/or miR-133, that resulted in human iCMs with gene expression profile, sarcomere structure, and spontaneous calcium transients reminiscent of cardiomyocytes (301–303). However, the use of viral vectors to ectopically express genes of interest finds a lot of skepticism. To eliminate this drawback, a virus-free method using a combination of nine chemicals was used to transdifferentiate human fibroblasts to cardiomyocyte-like cells that expressed cardiac genes and had a well-organized sarcomere structure. Even though it is not clear if the chemically reprogrammed cardiomyocyte-like cells underwent through a progenitor state, when they were transplanted into a murine heart after MI, they obtained a cardiomyocyte signature and were spontaneously beating (304). To eliminate the transplantation side effects, another strategy aiming to direct reprogramming *in vivo* was performed using the GMT and GHMT retroviral cocktails. Administration of the viral cocktail was performed directly in murine hearts after MI and lineage tracing of fibroblasts revealed that transduced resident fibroblasts were reprogrammed into cardiomyocyte-like cells. The reprogramming efficiency was lower than the one achieved *in vitro*. However, the *de novo* cardiomyocytes revealed similar behavior to endogenous cardiomyocytes which was better than that *in vitro* and resulted in reduced size of fibrotic tissue, implying the importance of the resident cardiac microenvironment (288, 294, 303, 305).

Obviously, the reprogrammed cells are new subjects in the cellular interactions governing the cardiac milieu. Thus, the better than expected *in vivo* performance of the reprogramming protocols may be owing to a paracrine action inhibiting resident cardiac fibroblast proliferation and associated fibrosis, promoting cardiac endothelial cell expansion (neoangiogenesis) or mediating cardioprotection by improving cardiomyocyte survival. In addition, as mentioned the inflammatory milieu of an injured heart may positively affect their engraftment, survival, and activity. However, inflammatory gene activation is not always beneficial as it appears to counteract adult cardiac fibroblast reprogramming (289).

### Manipulating the Immune Response

Despite earlier failures [reviewed in (306, 307)], a recent large scale (>10,000 participants) clinical trial targeting IL-1β secured that manipulating inflammation is a valid target in ischemic heart failure (308, 309). Based on their abundance, interaction with cardiomyocytes, and fibroblasts and their potent reparatory phenotypes assumed during ischemic HF progress (125, 131, 178), macrophages can play a leading role. Accordingly, liposome-mediated induction of anti-inflammatory properties of macrophages promoted angiogenesis and preserved cardiac structure and function in a rat model of acute MI (310). Macrophage status and maturity may also affect cardiac regeneration as deduced from comparisons between neonatal and adult mouse injury models (120, 164, 311), as well as the regenerating newt and zebrafish, but not medaka, teleost hearts (179, 216). The potential pro-and anti-regeneration abilities of the particular macrophage subsets populating the mammalian and, more importantly, human heart (125) in steady state and upon injury have not been presently conclusively mapped, but this is an obvious task.

Moreover, innate immunity activation can be used as a driving force to mobilize stem cells and regeneration [reviewed in (312)] and is actually required for successful nuclear reprogramming, at least in the iPSC protocols (313). In this context, in a recent cell therapy trial combined macrophage and mesenchymal stem cell cardiac delivery into ischemic HF patients achieved 37% reduction in adverse cardiac events (including deaths and hospitalization) (314). Furthermore, when macrophages were depleted from rats undergoing MI, the cardioprotective effect of cardiospheres, administered after the infarct formation, was abolished (315). In the same setting, the cardiospheres paracrinely modified macrophages to confer in turn antiapoptotic protection to cardiomyocytes.

Whether signals released by the stressed cardiomyocytes upon various insults that recruit macrophages and other inflammatory cells [reviewed in (316)] can be artificially oriented toward a beneficial outcome is of great importance, but such attempts should be planned with caution for several reasons. First of all, some of the native signals are actually beneficial. Upon MI, border zone cardiomyocytes express early cardiac transcription factors in a process reminiscent of dedifferentiation. They also secrete the small Reg3β protein to recruit reparatory macrophages, thus protecting the intact remote myocardium from damage by inflammatory neutrophils (317). Second, resident and recruited macrophages may considerably differ in their contribution to cardiac repair and regeneration in a time-dependent manner. For instance, in an animal model of non-ischemic HF resident macrophages initially support angiogenesis and repair but macrophages recruited subsequently are anti-angiogenic and detrimental (145). Furthermore, immunomodulation of cardiac repair and regeneration should be feasible, but must be timely and properly applied, as well as in harmonious conjunction to the parenchymal and stromal cardiac cells (4, 22, 118, 204). Apart from redirecting immune response by modifying additional pathways and populations such as that of mast cells, DCs, as well as components of the adaptive immune response [elaborated in (118)], beneficial outcomes may also take advantage of the cardiac lymphatic endothelium that seems to moderate MI inflammation (318).

## Conclusions-Perspectives

Recent advances enlightened important characteristics of cardiac cell populations and their mutual interactions, as well as the emerging opportunities to exploit these interactions toward efficient repair and regeneration following heart injury. In mammalian hearts, resident cardiac fibroblasts and macrophages and a permissive extracellular matrix sustain cardiomyocyte regeneration during a very short time window after birth. This opportunity is, however, lost later in life and minimal cardiomyocyte proliferation cannot support the loss of cardiac tissue after an injury. Whereas, resident macrophage subsets may sustain repair, mononuclear cell-derived macrophages, recruited in the heart by injured cardiomyocyte signals, may exert detrimental inflammation and polarize fibroblasts to promote cardiac fibrosis, hypertrophy, and further inflammation, building up a regeneration-hostile environment. Along with macrophages, additional immune cells become activated and involved mediating cardiac fibrosis and dysfunction in both acute and chronic injury and stress conditions. However, parts of the inflammatory response and its interactions with cardiac parenchymal and stromal cells are actually beneficial and necessary to sustain cardiac repair and regeneration following an insult or in interventional approaches.

Despite the tremendous research efforts and progress, human hearts remain unprotected after injury and insult. Any forced cardiomyocyte and progenitor mobilization attempts are of very limited efficacy and HF is still incurable with main medications targeting symptomatic neurohumoral changes and organ transplantation being the ultimate salvation tool. What is needed to achieve human cardiac repair and regeneration in the future? We hope it is obvious from the above that the answers to this central question will be multifaceted.

Basic research approaches combining Cre recombinase-based-or other genetic lineage tracing with single-cell transcriptomic and advanced proteomic and metabolomic analyses, should clarify the identities and qualities of the major cardiac cell populations and their timely changes following particular insults leading to HF. This is important and challenging given the plasticity of fibroblasts and macrophages and a certain degree of overlapping identities among cardiac cells (88, 100, 319). It is also expected to increase the probability of revealing novel target pathways and molecules for modifiable pro-regenerative protocols. It will be very crucial to find efficient ways to guide *in vivo* resident cardiac cells such as fibroblasts to transdifferentiate to a cardiogenic fate instead of a fibrotic one, thus accomplishing both decrease in fibrosis and more sufficient regeneration. Interventions targeting ECM appear to be particularly important for cardiac regeneration and repair. At the preclinical level, the new technologies using ECM scaffolds and injectable hydrogels are expected to further develop, aiming to improve the tissue reception of exogenous stem-or cardiac-committed cells. The latter appear to contribute more in a paracrine manner rather than by getting transformed to cardiomyocytes, thus analysis of their secretomes and efficient delivery are expected to improve repair. In addition, cross-species approaches such as administration of zebrafish ECM to murine hearts (320) provide useful links to successful pro-regenerative recipes. Furthermore, when manipulating fibrosis one should always keep an eye on its beneficial roles even for regeneration (217). Finally, exosome-, liposome- and nanoparticle-based advances in cell-free delivery technologies of therapeutic molecules (ranging from biologicals, antibodies, and antagonists to microRNAs, proteins, and even whole cell secretomes) are underway to more efficiently target myocardial tissue (4, 118, 204). Ideally, such approaches would combine pro-regenerative identities from all cell players, that is, native or engineered cardiomyocytes, fibroblasts, and cardiac macrophages, simultaneously excluding any of their counter actions that are known or will be revealed in the near future to inhibit regeneration. Furthermore, the mutual interactions of cardiomyocytes and the non-myocytes during HF progress should be carefully taken into account, to maximize the benefits of timely planned interventions. The success will probably lie in therapies simultaneously manipulating more than one cardiac or extracardiac population.

All these efforts attempt to either directly replenish the failing cardiomyocyte population or motivate endogenous pro-repair and pro-regenerative pathways in the human heart. The precise understanding of cardiomyocyte, stromal, and immune cell identities and interactions in steady state and at particular time points during the course of cardiac injury will allow our success in a currently unresolved health issue.

## Author Contributions

All authors listed have made a substantial, direct and intellectual contribution to the work, and approved it for publication.

### Conflict of Interest Statement

The authors declare that the research was conducted in the absence of any commercial or financial relationships that could be construed as a potential conflict of interest.

## Abbreviations

CF: Cardiac Fibroblasts
CS: Cardiospheres
CPC: Cardiomyocyte Progenitor Cell
CSC: Cardiomyocyte Stem Cell
CSP: Cardiac Side Population Cell
DAMP: damage-associated molecular pattern
DC: dendritic cell
ECM: Extracellular Matrix
EMT: endothelial-to-mesenchymal transition
HF: Heart Failure
HFpEF: HF with preserved in ejection fraction
iCM: Induced Cardiomyocyte-like Cell
iPSC: Induced Pluripotent Stem Cell
iPSC-CM: iPSC-derived cardiomyocytes
MI: Myocardial Infarction
MSC: Mesenchymal Stromal Cell
TAC: Thoracic Aortic Constriction.
